# Supplementary material for: Optimized In Vitro CRISPR/Cas9 Gene Editing Tool in the West Nile Virus Mosquito Vector, Culex quinquefasciatus
Source: Insects. 2022 Sep 19;13(9):856. doi: 10.3390/insects13090856 (PMC9502113; doi:10.3390/insects13090856)
Supplement: Supplementary file 1 [file insects-13-00856-s001.zip › insects-1905124-supplementary.pdf]

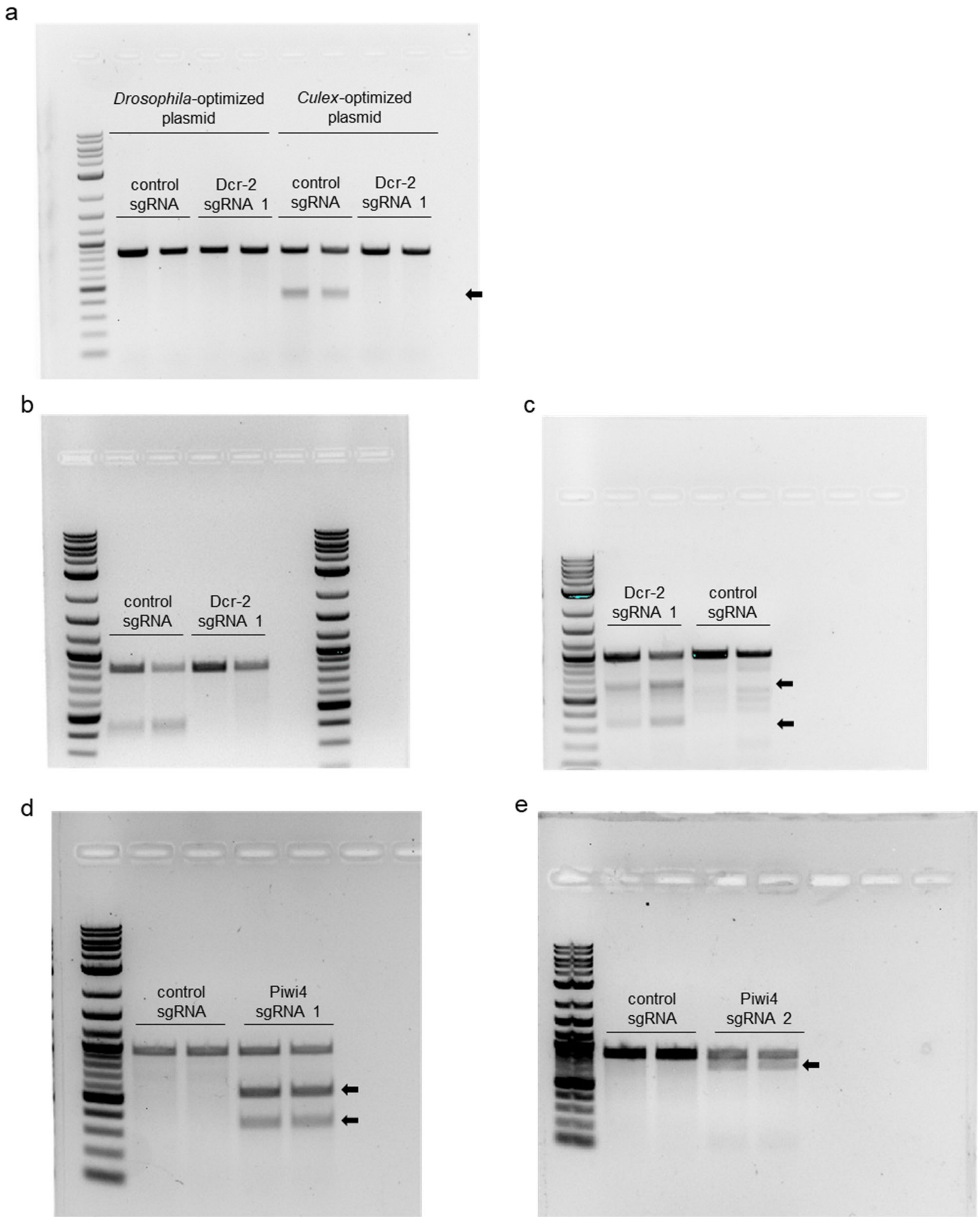

Figure S1. Original agarose gel electrophoresis images following T7 Endonuclease I (T7EI) assay used to generate main text Figure 3b (a), Figure 4 (b, c), and Figure 5 (d, e). Black arrows indicate T7-cleaved products.

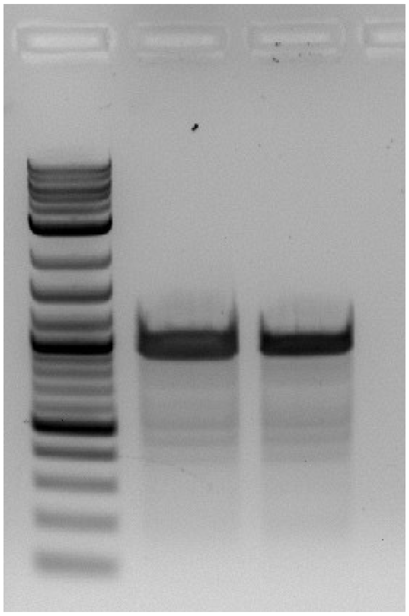

Figure S2. T7 Endonuclease I (T7EI) assay of the *dcr-2* genomic region targeted by sgRNA2 performed on untreated Hsu. Duplicate gDNA samples were used. Non-specific bands were observed, comparable to those in the 'control sgRNA' treated samples in Figure 4.
